# Use of TSAR, Thermal Shift Analysis in R, to identify Folic Acid as a Molecule that Interacts with HIV-1 Capsid

**DOI:** 10.1101/2023.11.29.569293

**Published:** 2023-11-29

**Authors:** X. Gao, W. M. McFadden, X. Wen, A. Emanuelli, Z. C. Lorson, H. Zheng, K. A. Kirby, S. G. Sarafianos

**Affiliations:** 1 Center for ViroScience and Cure, Laboratory of Biochemical Pharmacology, Department of Pediatrics, Emory University School of Medicine, Atlanta, GA; 2 Children’s Healthcare of Atlanta, Atlanta, GA

## Abstract

Thermal shift assay (TSA) is a versatile biophysical technique for studying protein interactions. Here, we report a free, open-source software tool TSAR (Thermal Shift Analysis in R) to expedite and automate the analysis of thermal shift data derived either from individual experiments or large screens of chemical libraries. The TSAR package incorporates multiple, dynamic workflows to facilitate the analysis of TSA data and returns publication-ready graphics or processed results. Further, the package includes a graphic user interface (GUI) that enables easy use by non-programmers, aiming to simplify TSA analysis while diversifying visualization. To exemplify the utility of TSAR we screened a chemical library of vitamins to identify molecules that interact with the capsid protein (CA) of human immunodeficiency virus type 1 (HIV-1). Our data show that hexameric CA interacts with folic acid *in vitro*.

## Introduction

The thermal shift assay (TSA), also called differential scanning fluorimetry (DSF), is an *in vitro* technique used to study the thermal stability of a purified protein or biological complex that has multiple applications [1–3]. During a TSA experiment, samples are heated to boiling temperatures at a constant rate to track the denaturation process of the protein(s) of interest. The denaturation of proteins that contain internal aromatic residues, like tryptophan, can be tracked without a dye by measuring the change in residue fluorescence [4]; however, TSA experiments often utilize a dye, like SYPRO^™^ Orange, to follow the exposure of the hydrophobic residues as denaturation progresses as the dye is fluorescent in hydrophobic environments [5]. Plotting the sample temperature compared to the fluorescence gives an individual melting profile for the protein or complex measured. The melting curve of an uncharacterized protein or complex can be unpredictable, and testing of various dyes will help validate the findings [6]. For folded and globular proteins, hydrophobic residues are typically internal and inaccessible to the solvent, making TSA a useful tool to assess if a batch of proteins is properly folded, with low initial fluorescence readings, or denatured after purification, with high initial fluorescence readings near room temperature [7, 8].

For most samples that are properly folded, the thermal profile is sigmoidal, and a melting temperature (T_m_) value can be calculated, representing the temperature where 50% of the protein is unfolded [2, 7]. The T_m_ is a useful value to compare the thermal stability of proteins under various conditions, such as choosing storage buffers and salts or for comparing the stability of mutant proteins [9, 10]. TSA is also useful for screening chemical libraries to identify potential binding partners for proteins, since protein•ligand interactions that form additional intramolecular bonds require more energy, or a higher temperature, to denature the sample compared to an unliganded protein [11, 12]. Thus, TSA has been used as a proxy readout for *in vitro* ligand binding within screens for protein inhibitors of a specific target.

**Figure 1 –:**
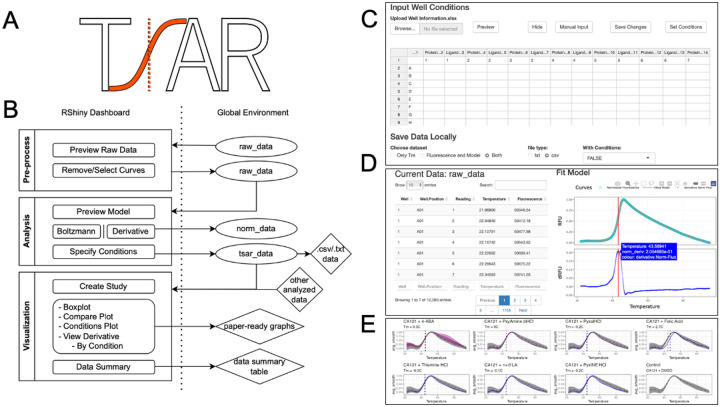
A) TSAR Logo. B) Workflow for TSAR, supporting data import and export at various stages. C-E) Screenshots of the TSAR program. C) shows the Shiny R GUI for importing data. D) shows the interactive data analysis that TSAR performs, E) is an example of a partial output from a library screen.

This technique is used extensively in labs that focus on biological research, as it requires only a qPCR machine for data acquisition once a protein or biological complex is purified [2, 6]. The required volume and concentration of the sample are quite low, typically under 25 μL and below 20 μM per sample, although multiple replicates are needed when using TSA for comparison and analysis. Present tools are Protein Thermal Shift^™^ Software v1.4 by Thermo Fisher Scientific (Waltham, MA), Serial Explorer in MATLAB, and open-source workflows provided through the Konstanz Information Miner (KNIME) [13]. While both tools from Thermo Fisher Scientific and MATLAB provide high-throughput analysis, they are proprietary and require additional costs. Despite KNIME being open source, it offers only limited visualization options. Given the need to visualize the data in multiple dimensions and enhance accessibility of analysis tools we developed a software package written in R, a programming language extensively used for statistical and graphical analysis of biological data. ‘TSAR’, short for Thermal Shift Analysis in R, is a free and open-source software operating in R (version ≥ 4.3). It is distributed under an AGPL-3 license that offers a TSA analysis workflow as both command-line functions and an interactive ShinyR graphic user interface (GUI) dashboard [14, 15] (Figure 1). TSAR analysis incorporates either Boltzmann-fit or derivative-modeling of data to identify either T_m_ B (Boltzmann-fit) or T_m_ D (derivative-model) from melting curves with pipelines for easy comparisons of numerous dimensions, including T_m_s, ligands, dosages, derivatives, and time series. The TSAR package is a complete suite of tools from initial data processing, T_m_ identification, automated data comparisons, and visualization of multiple publication-quality graphics for individual experiments to large screens with replicates (Figure 1B–E). TSAR is a well-documented package and has many custom settings for users experienced in R, while it also incorporates wrapping functions and a ShinyR dashboard to optionally minimize coding for easy use and quick implementation. Overall, TSAR aims to simplify TSA analysis yet diversify visualization.

Here, we perform an example TSA screen using the TSAR package to identify potential binding partners to the human immunodeficiency virus type 1 (HIV-1) capsid protein (CA). The HIV-1 capsid is an essential macromolecular structure within the mature viral core that serves as a reaction chamber for reverse transcription [16, 17]. It also protects the viral genome from cellular immune sensors as it traffics the genetic material to the nucleus [18, 19]. As it is important in numerous steps throughout the HIV-1 replication cycle, investigating the biology of the capsid core is of great importance in the design of next-generation antiretroviral therapies (ART) [18]. In fact, when the CA-targeting compound Lenacapavir (also called Sunlenca^®^ or GS-6207) was approved for use in highly-treatment experienced patients in 2022, CA became the first new target for a clinically used ART class in over a decade [20, 21]. CA is a great clinical target, as it is a highly conserved and genetically fragile protein; the kinetics of CA•CA interactions are finely tuned for balancing cellular trafficking and genome release, such that both increasing and decreasing capsid stability has a negative impact on viral fitness [9, 18, 22–25]. Recent reports have demonstrated that CA interacts with inositol hexaphosphate (IP6) and deoxyribonucleotides (dNTPs) to regulate and increase capsid stability [26–28]. As there are reports that HIV-1 infection modulates cellular metabolism, both in the presence and absence of ART, we hypothesized that there might be additional endogenous interactions with other common protein cofactors [29, 30]. Thus, we screened a library of essential vitamins (Sigma, St Louis, MO) for interactions with a disulfide-stabilized HIV-1 capsid hexamer and found that folic acid shifted the T_m_ D by 3.04°C. This screen provides an example workflow for the TSAR R package, a tool that can be broadly used in protein biology.

## Methods

### Distribution and Package Information

TSAR (Thermal Shift Analysis in R) is an open-source package written in R (≥4.3) [15]. It is freely distributed under an AGPL-3 license in the Bioconductor R package repository (V ≥3.18) [31] at https://bioconductor.org/packages/TSAR. A prerequisite for downloading TSAR through R requires that the BiocManager R package [32] be installed prior; installing TSAR is accomplished with the following line of code: *BiocManager::install(“TSAR”)*. TSAR was coded and documented using RStudio GUI [33] and the devtools package [34]. TSAR contains well-documented functions and three vignettes (long-form documentation) to assist users. TSAR depends on tidyverse packages [35] as well as: jsonlite [36], openxlsx [37], mgcv [38–40], and minpack.lm [41] packages for analysis, and shiny [14], shinyWidgets [42], plotly [43], shinyjs [44], rhandsontable [45], and ggpubr [46] packages for the Shiny R GUI and data visualizations.

### Package Structure

TSAR is sectioned into three individual operating dashboards, Data Preprocessing, Data Analysis, and Data Visualization, each with one command line call (Figure 1B). While users may conduct all procedures and analyses within the established GUI, this package also allows command-line approaches where users may implement more options and flexibilities with their analysis. Mathematical modeling in TSAR relies on both generalized additive models and non-linear modeling to compute T_m_ D and T_m_ B values.

TSAR allows visualization of data on multiple dimensions, taking T_m_ (50% protein unfolding temperature) analysis further, providing a comparison of numerous dimensions, including aspects of, but not limited to T_m_s, ligands, dosages, derivatives, and time series.

### Statistics and Analysis

**Figure 2 –:**
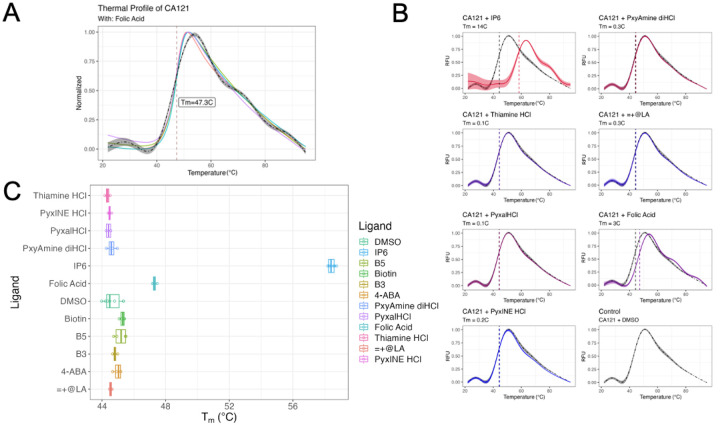
A) Compare plot further showcases the interaction curves. Graphed as RFU (relative fluorescence unit) over Temperature (degree Celsius) in color as contrast to DMSO control in grey. Only IP6, positive control, and Folic Acid yields significant T_m_ change. IP6: *Δ*T_m_ D = 13.75°C at significance level < 5E-11. Folic Acid: *Δ*Tm D = 3.04°C at significance level < 5E-5. B) Folic Acid thermal shift assay profiles by individual sample (n = 4). Each individual trial is mapped in a differently colored curve. Graph shows normalized RFU (relative fluorescence unit) as y-axis. Alternate options are allowed in TSAR package. The grey line represents average between all samples of a condition. C) Screening all vitamin compounds at 50 μM with CA121 hexamer protein at 7.5 μM. All compounds were suspended in DMSO, thus compared to negative control, DMSO. Initial screening shows that folic acid shows a 3.04°C of Tm D shift, indicating interactions with CA121 while it unfolds.

TSAs result in a standard curve characterized by initial low fluorescence, an increasing peak, and trailing fluorescence slowly decreasing back to the initial level. To study such peaks, mathematical modeling is required (Figure 2A). Combining with machine learning, TSAR utilizes the generalized additive model from mgcv package to capture the derivatives of the curve and the inflection point, T_m_ D [38–40]. Meanwhile, TSAR utilizes non-linear modeling from the minpack.lm package [41] in R to capture Boltzmann curves with machine-learned minimum and maximum. By this mechanism, TSAR is able to characterize the fluorescence trend in Boltzmann fit fashion, locating the T_m_ B. Both methods may switch between easily within the GUI and command line. To assess the accuracy of TSAR, we benchmarked its calculations of T_m_ and ΔT_m_ shift to those of Protein Thermal Shift Software V1.4 (Thermo Fisher Scientific). Setting Protein Thermal Shift Software calculations as the baseline literature value, the error of TSAR T_m_ D is centered at 0.048 with a variance of 0.063 degree Celsius (Supplementary Figure S1A). Meanwhile, the error of TSAR T_m_ B is centered at 1.029 with a variance of 0.634 degree Celsius (Supplementary Figure S1B). While that error is relatively greater than T_m_ D, the error is systematic where computing ΔT_m_ B by using DMSO as the baseline control yield errors centering at 0.376 °C (Supplementary Figure S1C–D).

**Figure 3.**
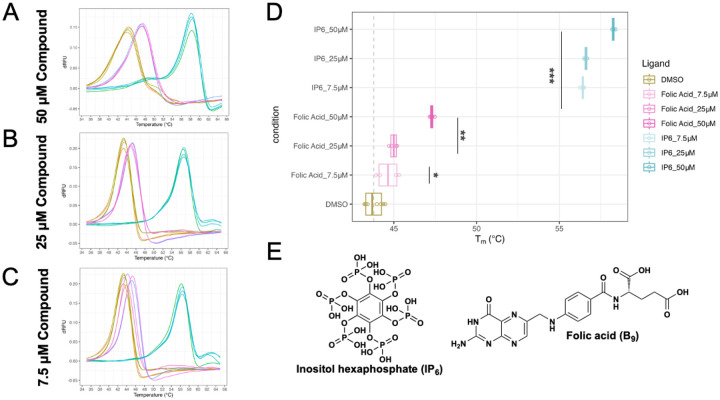
Dose dependence study of folic acid and CA121 shows *Δ*T_m_ is dosage-dependent yet still significant. A-C) First derivative comparison graphs demonstrate that *Δ*T_m_ of folic acid with CA121 is dose dependent at A) 50 μM, B) 25 μM, C) 7.5 μM compound. At 7.5 μM, protein unfolding curves are distinguishable, but suffers greater variance. D) Each condition (n = 4) consist of ligand suspended in DMSO and CA121 hexamer protein in Tris buffer at pH 8.0. IP6 is diluted in Tris directly without DMSO. One-tailed t-tests show that all treatment conditions are significantly different from the DMSO control. * indicates significance at 5E-3, ** indicates significance at 5E-5, and *** indicates significance at 5E-9 E) Chemical Structures of hits, Folic Acid and IP6. Made in ChemDraw V19.0.

Testing the library yielded significant result for the folic acid with *Δ*T_m_ D shift of 3.04 °C. Testing at 50 μM, 25 μM, and 7.5 μM compound, the first derivative comparison graphs demonstrate that the *Δ*T_m_ of folic acid with CA121 is dose dependent (Figure 3). At 7.5 μM, protein unfolding curves are distinguishable from DMSO control, but *Δ*T_m_ experiences greater variance. Conducting one-tailed, paired T tests show that all treatment conditions are significantly different from the DMSO control, with 50 μM and 25 μM folic acid significant at the level of 5E-5, and 7.5 μM folic acid significant at the level of 5E-3 (Figure 3D).

### Protein Purification

The CA hexamers with A14C/E45C/W184A/M185A mutations (CA121) were cloned into a pET11a expression plasmid, which was provided by Dr. Owen Pornillos from the University of Virginia. The expression and purification of CA121 were carried out in *E. coli* BL21(DE3)RIL as previously described [47]. Additionally, full-length, wild-type CA monomers (CA FL) were cloned into a pET11a expression plasmid, provided by Dr. Chun Tang from Peking University. The expression of CA FL was performed in *E. coli* BL21(DE3)RIL and purification involved ammonium sulfate precipitation followed by anion exchange chromatography, as previously described [25].

### Compounds

Library chemicals were purchased as part of the Vitamins Kit (Sigma Aldrich, V1–1KT) and resuspended in DMSO to a final concentration of 10–50 mM and stored at −20°C. Compounds included: D-Pantothenic acid hemicalcium salt (B5), Biotin, Nicotinamide (B3), 4-Aminobenzoic acid (4-ABA), Pyridoxamine dihydrochloride (PyxAmine diHCl), Pyridoxine hydrochloride (PyxINE HCl), Thiamine hydrochloride (Thiamine HCl), Folic Acid (B9), (±)-α-Lipoic acid (a-LA), Pyridoxal hydrochloride (Pyxal HCl). 50% Phytic acid solution (Sigma Aldrich) was used as the IP6 positive controls.

### Thermal Shift Assay (TSA)

TSAs were conducted using QuantStudio 3 Real-Time PCR Systems (Thermo Fisher Scientific, Waltham, MA) with a final reaction volume of 20 μL and ≤1% DMSO. The sample fluorescence was measured while being heated from 25–95°C at a constant rate of 0.2°C/10s, consistent with the methodology outlined in previous studies [6, 9, 11]. Proteins were tested at a final concentration of 7.5 μM in a 50 mM Tris (pH 8.0) and supplemented with 1x SYPRO^™^ Orange Protein Gel Stain (Life Technologies) after incubation with the compounds for 30 minutes on ice. In the initial screening of the library, the CA121 protein (7.5 μM) was incubated with 25 μM of each compound. Fluorescence intensity for each sample was measured as described, and thermal profiles were assessed using TSAR or Protein Thermal Shift Software v1.3 (Applied Biosystems) (Figure 2B–C). The compounds that induced a shift in the T_m_ (ΔT_m_) at 25 μM underwent further testing in a dose-response manner under concentrations of 7.5 μM and 50 μM (Figure 3).

**Figure 4.**
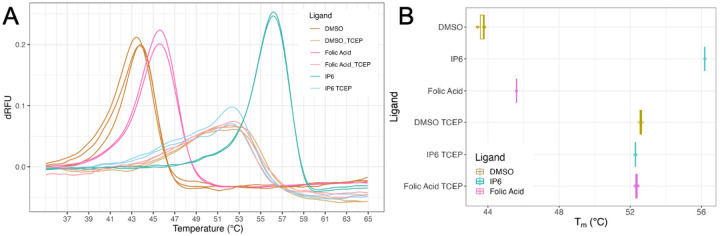
Reducing CA121 shows Thermal Shift in folic acid is protein specific. TCEP was added as a reducing agent to break apart CA121, eliminating the central pore of protein-ligand interaction. A) TSA performed with ligands at 25 μM. Comparing first derivatives show that TCEP alters regular protein fluorescence curve. B) Boxplot demonstrates adding TCEP leads to insignificant *Δ*T_m_ D.

Lastly, all compounds leading to significant thermal shifts were also tested in the presence of the tris(2-carboxyethyl)phosphine (TCEP)-reducing agent. TCEP functions to break the CA121 disulfide stabilizing bonds, possibly affecting the stability of pockets formed by multiple CA monomers [26]. Comparing the fluorescence profile of TSA samples with and without TCEP provides insight into the potential interactions of a compound with the monomer versus the hexamer CA protein (Figure 4).

## Discussion

Here, we performed a screen of protein•ligand interactions by conducting a thermal shift assay (TSA) and analyzed the results with the open-source R package, TSAR. We found that the HIV-1 capsid protein hexamers interact with folic acid *in vitro*. TSAR is a novel package for the R coding language, which has become a common tool taught in higher-education coursework and used by many biological research labs. There are other software tools for TSA data analysis, like the proprietary software offered by Thermo Fisher Scientific or open-source packages offered for the proprietary MATLAB software. However, these typically have some cost associated with implementing or installing the software. TSAR is written in the R coding language, which is free, and distributed under an AGPL license, ensuring there is no cost for analyzing the data.

There are reports identifying endogenous metabolites as important cofactors in HIV-1 replication that interact specifically with mature capsid hexamers [47], including IP6 that is important for capsid assembly and stability as well as dNTPs that are needed for reverse transcription and genome replication [16–19, 26, 27]. This study does not establish the biological significance of folic acid for the replication cycle of HIV-1. Of note, many individuals living with HIV-1 have a deficiency in folic acid metabolism [48, 49], though it is not clear these findings are related. Folic acid is an important factor required for cell growth and development; folic acid deficiency can lead to neuronal defects and anemia [50, 51]. Thus, it is of interest that this critical cellular metabolite may interact with and stabilize HIV-1 capsid hexamers. The role and relevance of folic acid in HIV-1 biology remains a topic of investigation, but the findings of the screen reported here indicate the ability for assembled hexamers, but not unassembled monomeric capsid, to interact with folic acid.

## Supplementary Material

1
