# Supplementary material for: Use of TSAR, Thermal Shift Analysis in R, to identify Folic Acid as a Molecule that Interacts with HIV-1 Capsid
Source: bioRxiv. 2023 Nov 29:2023.11.29.569293. Preprint. [Version 1] doi: 10.1101/2023.11.29.569293 (PMC10705415; doi:10.1101/2023.11.29.569293)
Supplement: 1 [file NIHPP2023.11.29.569293V1-supplement-1.pdf]

bioRxiv preprint doi: https://doi.org/10.1101/2023.11.29.569293; this version posted November 29, 2023. The copyright holder for this preprint (which was not certified by peer review) is the author/funder, who has granted bioRxiv a license to display the preprint in perpetuity. It is made available under aCC-BY 4.0 International license.

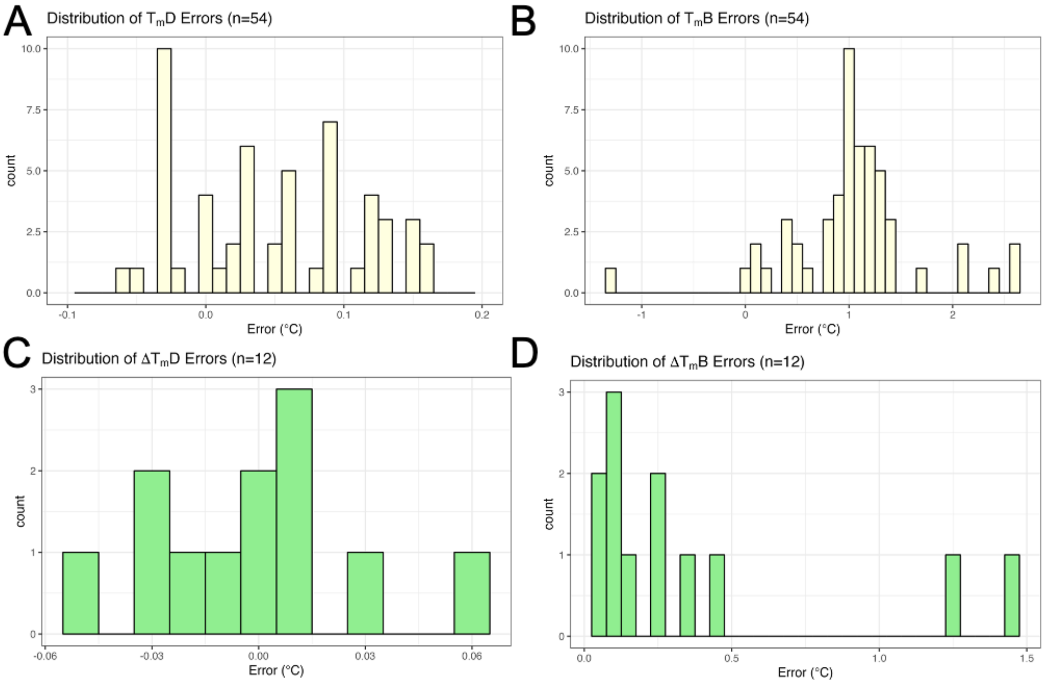

Supplementary Figure 1. TSAR accuracy compared to Protein Thermal Shift™. A, C) Derivative estimation errors. (n = 54) is N~(0.048, 0.063). ΔT_m D estimation error is similarly distributed, N~(-0.001, 0.029). **B, D)** Boltzmann estimation errors (n = 54) is approximately N~(1.029, 0.634). ΔT_m B estimation error is biased in the positive direction, N~(0.376, 0.451).
